# Near-Zero Temperatures Arrest Movement of the Diaheliotropic *Malva sylvestris*

**DOI:** 10.3390/plants12132484

**Published:** 2023-06-29

**Authors:** Elena Arvaniti, Efi Levizou, Aris Kyparissis

**Affiliations:** 1 Department of Biological Applications and Technology, University of Ioannina, 451 10 Ioannina, Greece; arvaniti.elena82@gmail.com; 2 Department of Agricultural Crop Production and Rural Environment, University of Thessaly, Fytokou Str., 384 46 Volos, Greece; elevizou@uth.gr

**Keywords:** common mallow, solar tracking, Ψ, photoinhibition, leaf inclination, azimuth, cos(i)

## Abstract

In the present study, the diaheliotropic leaf movement pattern of *Malva sylvestris* in relation to the impact of low temperature is presented. Seasonal measurements of movement characteristics along with important aspects of plant function, such as chlorophyll content, water potential, PSII photochemistry, and phenological parameters were performed on plants in their natural environment. During the study period, low winter temperatures and a 10-day freezing event gave insights into the plant’s response to harsh environmental conditions and the effect of the latter on leaf movement profile. Plant growth was significantly inhibited during low-temperature periods (leaf shedding) and the photosynthetic performance was seriously depressed, as judged by in vivo chlorophyll *a* fluorescence. Additionally, the diaheliotropic leaf movement pattern was arrested. Temperature rise in March triggered new leaf burst and expansion, enhancement of the photosynthetic performance, and the recovery of the diaheliotropic movement. The daily and seasonal profiles of the water potential were synergistically shaped by leaf movement and climatic conditions. We conclude that diaheliotropism of *M. sylvestris* is a dynamic process that coordinates with the prevailing temperatures in ecosystems like the studied one, reaching a full arrest under near-zero temperatures to protect the photosynthetic apparatus from over-excitation and prevent photoinhibition.

## 1. Introduction

Plants are considered immobilized organisms; however, they can exhibit a wide variety of movements, ranging from the sub-cellular to the organ level [1]. One of the earliest recorded observations of leaf movements was made by Theophrastus, a Greek philosopher and botanist who lived in the 3rd century BCE, in his work “Historia Plantarum”. About 2200 years later, in the 19th century, it was Charles Darwin and his son Francis with the book “The Power of Movement in Plants” who paved the way for understanding the leaf movements and their underlying mechanisms, by conducting extensive experiments in the movements of sunflowers and other species [2]. Leaf heliotropism is the most-studied plant movement and refers to the ability of leaves to track the sun’s movement across the sky during the day, while completely resetting leaf position during the night [3]. There are two types of leaf heliotropism; diaheliotropism, in which leaves are oriented perpendicular to the sun’s rays in order to maximize light absorption, and paraheliotropism, in which leaves are oriented parallel to incoming rays to minimize exposure to excess light.

All types of leaf heliotropism ultimately result in the regulation of the incident photon flux density at the leaf plane, which is directly related to photosynthetic and transpiration rates, water status, and thermoregulation needs [4,5,6]. Through modulating vital plant processes, heliotropism may be considered a light and temperature optimization mechanism [7,8]. This conceptual framework of leaf movements was substantiated by recent findings of complex and co-existing dia- and paraheliotropic movements in the same plant, and the multiple components of environmental control over leaf movements. Concerning the former, it was traditionally believed that plant species display a uniform and constant heliotropic pattern for all their leaves. In fact, some plants do that, but there is growing evidence that several others exhibit a more complex movement pattern. Recently, the complicated pattern of *Capparis spinosa* L. was revealed, in which the type of leaf movement differentiates with stem azimuth, leaf position on the stem, and time of day [9]. A shift from diaheliotropic movements in the forenoon to para- at midday is considered an effective adaptation strategy of *Sophora alopecuroides* to arid riparian ecosystems [10]. The non-leguminous *Styrax camporum* face the adverse environmental conditions of the Brazilian savanna by possessing two distinct leaf groups, namely the paraheliotropic ones that possess a certain position in woody stems and primary branches, and the diaheliotropic ones occupying other stems [11].

The above-mentioned and other documented combinations of movements are considered specific acclimation responses of plants to the prevailing environmental conditions. Soil water deficit [5,12], high photon flux density [6,13], high temperature [14], and low nitrogen availability [15] have been reported to positively influence the occurrence and degree of paraheliotropism. Under these stress conditions, excess light aggravates photoinhibition of photosystem II (PSII), thus steeper leaf angles confer photoprotection through the avoidance of the additional high-light stress (high energy load) [6,16]. It is estimated that a decrease of 40–70% in the incident light on leaf lamina and 5–10 °C lower leaf temperature is evident in light-avoiding leaves compared with restrained ones [8]. The protection of photosynthetic apparatus from the photo-damage imposed by water stress in soybean is ascribed to paraheliotropic movement-assisted dissipation of excess excitation energy resulting in the downregulation of PSII [17]. Likewise, Huang et al. [18] provide experimental evidence that the light-avoiding movements of *Bauhinia tenuiflora* are regulated by the PSII activity, with photoinhibition playing a critical role. Overall, paraheliotropism is considered a stress-alleviating mechanism that may remedy the deficiency of photoprotection capacity in the relevant plant species.

The ecophysiological significance of diaheliotropism mainly lies in the improved carbon gain integrated over the course of the day [19,20,21]. Photosynthesis is particularly enhanced in the morning and afternoon hours when plant water status is more favorable and solar elevation is lower, making sun tracking more critical for enhancing available light [22]. Even though the benefits in daily productivity may be small, integrating them over the length of a growing period results in improved biomass accumulation. This advantage may be of particular value in ephemeral and annual vegetation that has to complete its life cycle in a limited time before the unfavorable season onset [22]. Likewise, solar tracking is a crucial factor contributing to increasing the productivity of crops such as cotton, given the optimal nutrient and water conditions ensured in intensive agriculture [17,19]. Diaheliotropic movements are performed by high-light demanding plant species with a specific suite of physiological characteristics, such as high photosynthetic light saturation points, high intrinsic photochemical efficiency of PSII [21], and effective photoprotection mechanisms including thermal energy dissipation and photorespiration [19], as well as other non-photochemical quenching processes [23]. The relevant literature, although detailed on the characteristics of leaf movements and photochemical performance responses, is very scarce on the influence of concurrent stresses [5,24]. Therefore, we have plenty of information on how diaheliotropic leaves cope with excess energy, but we know little about how adverse environmental conditions impact the pattern of leaf movement and induce potential modifications of it.

*Malva sylvestris* is a widely distributed herbaceous species, which occupies open and high-light habitats [25]. It bears the typical characteristic of the *Malvaceae* family, i.e., diaheliotropism, however, no reports on that feature have been published yet. The movement pattern of other congeneric species, such as *M. neglecta* and *M. parviflora* [26,27], as well as other members of the *Malvaceae* family, namely *Malva multiflora* (synonym *Lavatera cretica*) and the desert annual *Eremalche rotundifolia* (synonym *Malvastrum rotundifolium*) [28,29] has been extensively described in earlier studies. Even for these well-studied species, there is a lack of information about the influence of environmental factors other than light on the heliotropic movements.

Considering that abiotic stress is the rule and not the exception in plants’ life, the void of information on how stress modifies leaf heliotropic patterns is surprising. Given the dynamic nature of heliotropism as demonstrated by the above-mentioned examples of complex movement patterns, a strong response to stress is to be expected. Accordingly, the aim of the present work was to study the diaheliotropic movement pattern in *M. sylvestris* (common mallow) in relation to temperature and to explore its ecophysiological significance over a long time period of 10 months during which the plant experienced high to freezing temperatures. We have chosen to perform our study in a cold habitat of the common mallow, near the northern edge of its distribution in Greece, to ensure a naturally derived and long-term cold acclimation of the species. The leaf movement pattern was followed throughout the study period, along with several functional parameters to evaluate the combined effects of temperature and leaf movement on key physiological processes.

## 2. Materials and Methods

### 2.1. Species and Study Site

*Malva sylvestris* L. is a herbaceous annual, biennial, or perennial plant, native to Europe, Northern Africa and Southwestern Asia. Three naturally occurring individuals located in the open field under full sunlight conditions inside the campus of the University of Ioannina (39.62 N, 20.84 E) were selected for all field measurements, which were performed between October 2004 and July 2005.

### 2.2. Field Measurements

Leaf area was estimated from length and width measurements with the use of a standard curve. The standard curve was constructed by measuring length, width, and area in leaves of adjacent individuals covering a wide range of leaf sizes (area = 1.044 + 1.121 × a × b, where a and b are length and width in cm, r^2^ = 0.95). 

Leaf inclination (the angle between the lamina plane and the horizontal) and leaf azimuth (the angle between the lamina plane and the north) measurements were performed in 30 fully exposed tagged leaves (10 leaves per plant) in order to fully describe the movement pattern. After the freezing episode in early February 2005 (see results), new leaves were tagged and used in the subsequent measurements. Both leaf inclination and azimuth were measured as described in [9], following the methods originally developed in [30,31]. Solar elevation and azimuth data for the measurement days were downloaded from https://www.sunearthtools.com/dp/tools/pos_sun.php, accessed on 19 December 2022. All the above data were introduced to Equation (1) to calculate according to [30] the cosine of the angle of incidence (cos(i)) between the leaf plane and the sun’s direct beam, which represents the proportional incidence of direct solar beams upon a leaf [30,31].

cos(i) = cos(β) cos(z) + sin(β) sin(z) cos(α_s_ − α_l_), (1)

where β is the leaf angle from the horizontal, z is the solar zenith angle, α_s_ is the solar azimuth angle and α_l_ is the leaf azimuth angle.

The values of cos(i) may vary between 1 (adaxial leaf surface perpendicular to direct sun beam) and −1 (adaxial leaf surface not facing direct sunbeam), with values around 0 representing a leaf parallel to direct sun beam [32]. Measurements were performed at approximately 2 h intervals, from dawn to sunset.

Chlorophyll content was measured in the same tagged leaves used for movement measurements, with a CCM-200 chlorophyll content meter (Opti-Sciences, Inc. Hudson, USA). CCM data were converted to chlorophyll content (μg cm^−2^) with the use of a standard curve, which was constructed by measuring chlorophylls both with CCM and spectrophotometrically, in leaves of adjacent individuals covering a wide range of chlorophyll content (Chl (μg cm^−2^) = 18.443 + 0.629 CCM, r^2^ = 0.82). For spectrophotometric measurements, leaves were cut, sealed in airtight plastic bags, transferred to the laboratory, and used immediately. Chlorophylls were extracted with 80% *v*/*v* acetone and estimated using a U-2800 double beam UV–VIS spectrophotometer (Hitachi, Tokyo, Japan), according to Lichtenthaler and Wellburn [33].

Chlorophyll a fluorescence parameters, leaf temperature, and photosynthetic active radiation (PAR) were measured in vivo with a PAM-2100 pulse-amplitude portable modulated fluorimeter equipped with a 2030-B Leaf-Clip Holder (Walz, Effeltrich, Germany). PamWin software for PAM-2100 (v1.17) was used to extract chlorophyll fluorescence parameters, such as Fv/Fm, φPSII, and electron transport rate (ETR). Fluorescence measurements were performed at both predawn (naturally dark-adapted leaves) and noon (light-adapted leaves) on the same day, on the same plants/tagged leaves used for the previous measurements. The Fv/Fm parameter was derived from the pre-dawn measurements, while φPSII was measured at the light-adapted state at noon [34]. ETR values correspond also to the light-adapted state of mallow leaves.

All measurements were completed within 1–2 s and were performed at the natural orientation of each leaf. The recordings of the instrument’s PAR sensor (measuring at leaf level) and leaf temperature sensor were used for describing leaf light and temperature environment, respectively, over the course of the day. 

Leaf water potential (Ψ) was measured at predawn, midday, and afternoon at three similar nearby individuals. Measurements were performed in the field with a portable Scholander-type pressure chamber (SKPM 1400–80, Skye Instruments Ltd., Llandrindod Wells, UK), with a −8 MPa measuring limit. For each measurement, one or two randomly selected leaves per plant were wrapped in aluminum foil, sealed in plastic bags, and after 10 min were cut and measured immediately. 

Meteorological data (average daily temperature and total daily precipitation) for the study period were recorded by an automated meteorological station situated in close vicinity to the study site.

## 3. Results

**Figure 1:**
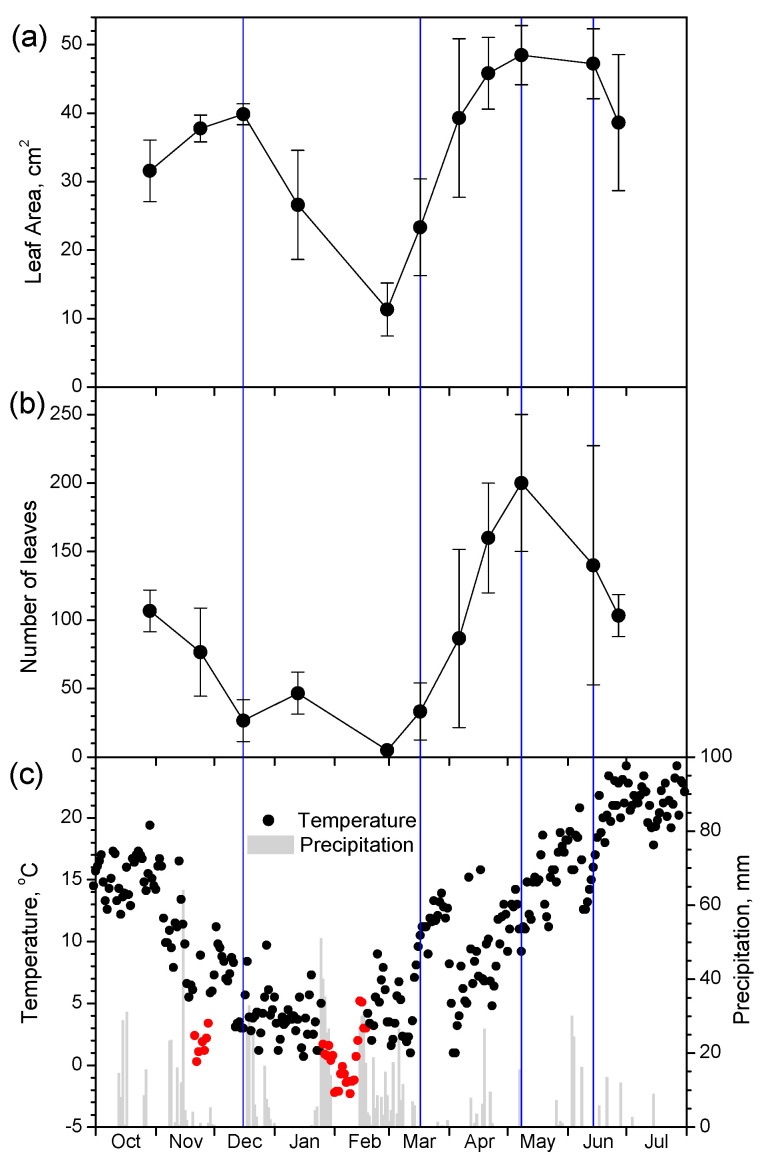
Seasonal fluctuation of leaf area (**a**), number of leaves per plant (**b**), and mean daily temperature and total daily precipitation (**c**) during the study period. Data in (**a**,**b**) are means ± SD from 3 plants. Blue vertical lines indicate dates with full diurnal leaf movement measurements. Red dots in (**c**) indicate freezing temperatures.

In Figure 1, the seasonal profile of the leaf area and number of leaves is presented together with the average daily temperature and total daily precipitation. During autumn, plants possessed a large number of mature leaves (area > 30 cm^2^). However, as the temperature gradually dropped during late autumn and winter and especially after periods of near-zero or below-zero temperatures (red dots in Figure 1c), the number of leaves decreased. Especially after the early February low/freezing temperature period, almost all leaves have fallen and only smaller, young leaves existed on the plants. As the temperature increased during the end of winter and spring, new leaves sprouted and expanded, increasing their area. At the end of spring, some leaves senesced, mainly the large ones, resulting in a decrease in the mean leaf area. 

**Figure 2:**
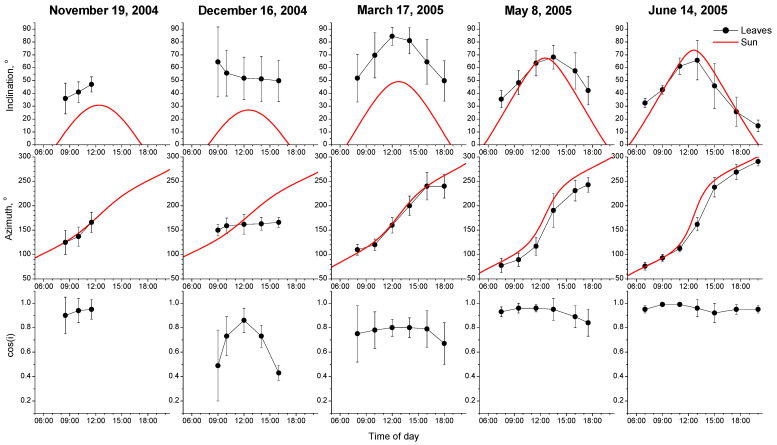
Diurnal course of leaf and sun movements for five dates that are indicated on top of each column. The first measurement (19 November 2004) is incomplete due to the appearance of clouds. First row: leaf and sun inclination; 0° corresponds to leaves vertical to the ground or sunbeams parallel to the ground and 90° to leaves parallel to the ground and sunbeams vertical to the ground. Second row: leaf and sun azimuth; 90° indicates leaves facing east (adaxial side) or sunbeams coming from east, 270° corresponds to leaves facing west (adaxial side) or sunbeams coming from west. Third row: cosine of incidence (cos(i)) between the leaf plane and a normal to the sun’s direct beam; values close to 1 correspond to leaves with their adaxial surface perpendicular to the sun’s rays, whereas values close to 0 correspond to leaves parallel to sun rays.

Even though seven diurnal measurements for leaf movement were attempted, only four of them were completed, due to the appearance of clouds during the course of the day (Figure 2). However, incomplete measurements during autumn (19 November) indicated that leaves were following sun movement, with a cos(i) pattern similar to that of spring (Figure 2). After the period of near-freezing temperatures during winter (Figure 1c, red dots) the leaf movement was canceled, since both leaf inclination and azimuth showed steady-state values throughout the day (16 December, Figure 2). As the temperature was rising during spring, the leaf movement pattern was restored partially, as indicated by the steady cos(i) values around 0.75 throughout the day on 17 March (Figure 2). Even though leaves’ inclination showed large changes during the day, there is a difference of about 30 to 40° between leaves and the sun, while azimuthal leaf movement closely follows sun azimuth change. At mid to end of spring (8 May and 14 June) leaves followed a fully diaheliotropic movement pattern, with both leaf inclination and azimuth closely following the sun and cos(i) remaining at steady values above 0.9 throughout the day.

**Figure 3:**
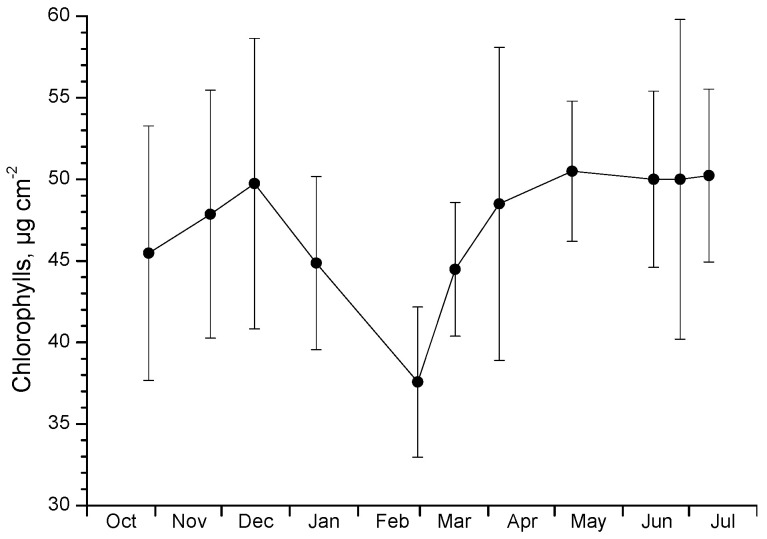
Seasonal course of total chlorophyll content. Data are means ± SD from 3 plants (10 leaves per plant).

**Figure 4:**
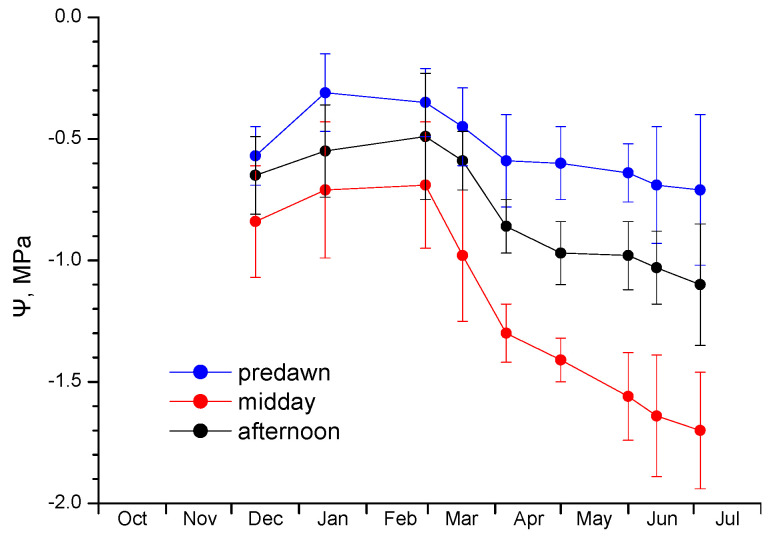
Seasonal course of predawn, midday, and afternoon water potential. Data are means ± SD from 3 plants (1 to 2 leaves per plant).

**Figure 5:**
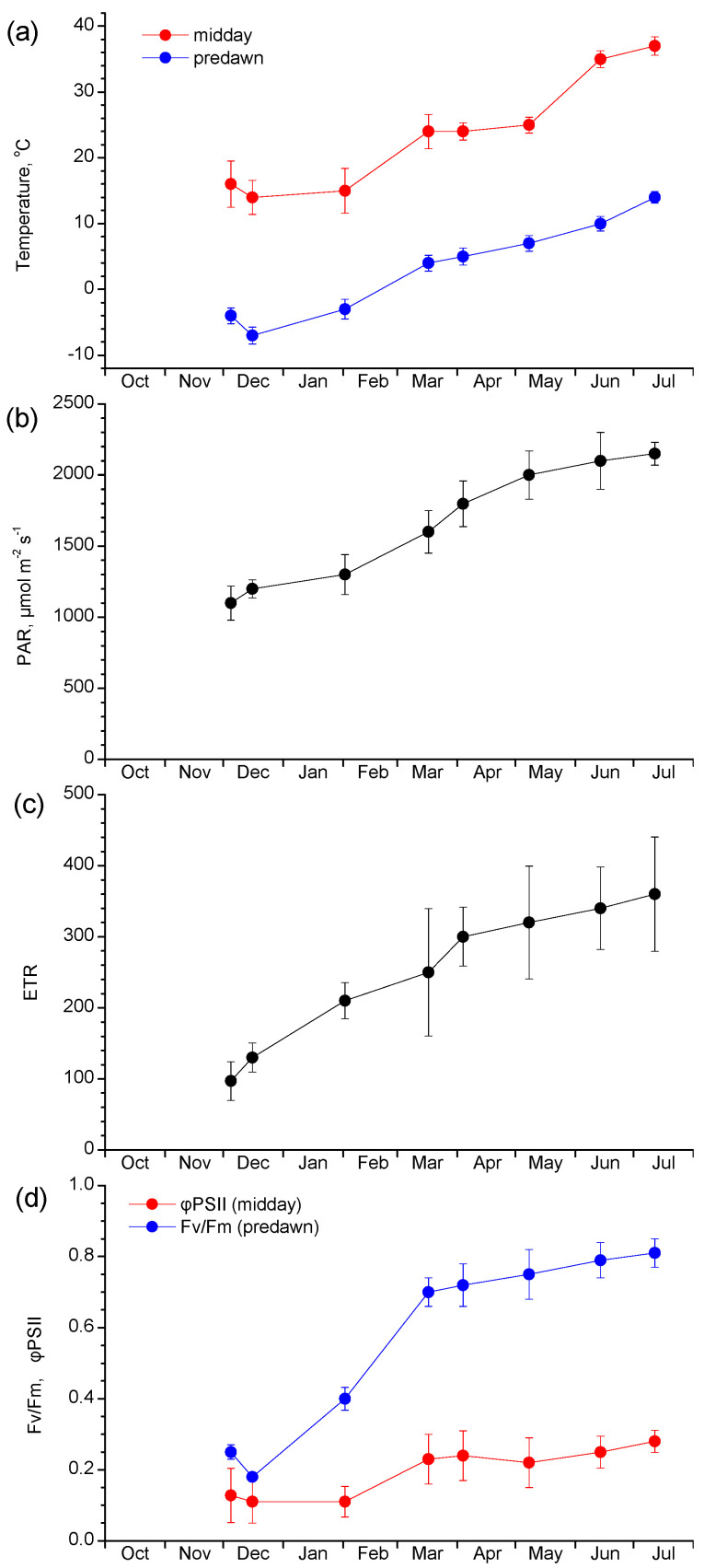
Seasonal course of leaf temperature (**a**), intercepted PAR at midday (**b**), midday ETR (**c**), and predawn Fv/Fm and midday φPSII (**d**). Data are means ± SD from 3 plants (10 leaves per plant).

Since the main advantage of diaheliotropism for plants is the maximization of the intercepted radiation resulting in higher daily photosynthetic capacity, photosynthetic performance was followed seasonally by measuring chlorophyll fluorescence at predawn and midday. Accordingly, leaf chlorophyll content and water potential were also measured as indicators of the photosynthetic apparatus status and possible stress conditions, respectively. As shown in Figure 3, chlorophyll content was steady throughout the season, except for the post-freezing February—March period, when most of the leaves fell and new ones emerged. Consequently, with the exception of the freezing period, plants did not seem to appear any serious damage that would have led to chlorophyll bleaching. Accordingly, water potential showed high values during winter and spring and a moderate depression at the end of spring–early summer (Figure 4). This finding indicates that no physiological water stress occurred during winter, even in cases of sub-zero predawn (minimum) leaf temperatures, as long as the midday (maximum) temperatures were above 10 °C (Figure 5a). 

Even though, as described above, no serious damage can be detected during the low-temperature winter period before the freezing event, the chlorophyll fluorescence pattern revealed dysfunction of the photosynthetic machinery. This is evidenced especially by the low predawn Fv/Fm values during December and February (Figure 5d). The pre-dawn Fv/Fm is a robust indicator of the maximum efficiency of PSII photochemistry, with values of non-stressed, normally functioning leaves ranging between 0.75–0.85 [34]. Consequently, the values below 0.3 (December) or around 0.4 (February) determined in mallow leaves indicate a negatively affected PSII performance probably connected to the prevailing sub-zero predawn leaf temperature (Figure 5a). Conversely, in the spring period as leaf and air temperatures rose well above zero, the functionality of the photosynthetic machinery was restored, resulting in high values of Fv/Fm (0.70–0.75). A similar seasonal profile appeared in the midday φPSII (Figure 5d). φPSII measured at the light-adapted state at noon indicates the PSII operating efficiency in the light. Again, during the cold December to February period, the lowest φPSII values were recorded (0.11–0.12), corresponding to the reduced quantum efficiency of PSII e^-^ transport under the ambient light. φPSII recovered in the spring and summer months, reaching its maximum value (0.28) during July. The ETR (Figure 5c), which is indicative of the rate of electron transport through PSII, incorporates the changes in both the light environment and the performance of the photosynthetic machinery. Consequently, the ETR profile (Figure 5c) closely followed both the PAR (Figure 5b) incident to leaf and φPSII, and presented a pronounced uptrend after the end of the cold winter period, reaching high values from the March measurement onwards.

## 4. Discussion

The growth profile of common mallow closely followed air temperature changes throughout the October to July study period. The plants possessed many large mature leaves during autumn with mild temperatures, which started to shed in mid-December as the temperature gradually dropped. After the freezing event in early February which lasted for 10 days, both the number and area of the remaining leaves reached their minimum. After this time point when the plants bore few and small young leaves, temperature rise at the beginning of spring triggered new leaf burst and expansion. Summer high temperatures bringing the onset of the dry period were marked by a partial leaf drop, mainly of the large leaves.

The chlorophyll content of the mallow leaves presented a slight decline after the mid-December measurement, which was exacerbated by the freezing event. As a result, a minimum was reached by the end of February corresponding to 25% lower chlorophylls in comparison with December levels. It has to be noted here that this decline in chlorophyll profile at the plant level is related to the age of leaves measured on each date. As mentioned in the growth-related results, after December and, moreover, after the freezing event, plants bore only new leaves as the mature ones had fallen. The chlorophyll content of these new leaves is lower than the previous mature ones, therefore, the reduced chlorophyll concentration in winter may be rather ascribed to developmental reasons, representing an age effect. Nevertheless, decreases in chlorophyll content due to low temperatures have been regularly reported either as stress-induced suppressions of chlorophyll biosynthesis or as an acclimation mechanism in cold-tolerant species [35,36]. A rise in mallow chlorophyll concentration was evident during spring when temperature levels were optimum and were kept steadily high in early summer. Muller et al. [37] reported that winter leaves of the congeneric *M. neglecta* contained higher chlorophyll content compared to summer leaves. However, in another study with below-zero temperature stress imposed on the same species, no apparent drop in chlorophylls was found [38]. 

The detailed description of the daily and seasonal profile of Ψ reveals the differential influence of temperature in Ψ during the course of the day, in close relation to the diaheliotropic habit. Pre-dawn Ψ appeared to be virtually stable throughout the study period, with only small deviations towards higher values in mid-winter and lower ones during spring and early summer. This finding reflects the presence of ample water reserves in the soil during the winter period that are partially retained in spring, supporting the recovery of the plant’s water potential during the night. Afternoon Ψ showed a similar profile, however, the downward trend in the spring–summer period was more obvious. As expected, the midday Ψ was always lower compared to pre-dawn and afternoon values, but its decline during March and onwards is more pronounced and steeper. Probably, the higher spring and summer temperature and the continuous sun-tracking, as judged by the movement pattern characteristics presented in Figure 2, synergistically shape the midday Ψ profile. In their early work on the ecophysiology of the desert annual *E. rotundifolia* (synonym *M. rotundifolium*) (Malvaceae), Forseth and Ehleringer [29] found that solar tracking movement is maintained even at very low water potentials, albeit at decreased stomatal conductance, due to osmotic potential changes that enable the maintenance of positive turgor. The minimum Ψ with partially open stomata recorded in this study was −3 MPa, far lower than in our case (−1.7 MPa).

The movement pattern of *M. sylvestris* in the present work was predominately influenced by the temperature of the preceding period. A typical diaheliotropic movement pattern has been recorded during May and June. The same was also observed during incomplete measurements of autumn and several other spring days, without being possible to be properly recorded due to cloudiness; however, the whole-day movement profiles presented in spring measurements of Figure 2 are absolutely representative of these incomplete measurements. In these time points, leaf inclination, i.e., the angle of the lamina with the horizontal plane, tightly followed the sun inclination with slight deviations very early in the morning and very late in the afternoon. Similarly, leaf azimuth, i.e., the angle with the magnetic north, did not diverge from the solar one by more than 30° in May, which was evened in the summer measurement. The combined effect of the above was the steadily high values of cos(i) above 0.9, thus near the maximum. Since cos(i) is a measure of the proportion of direct sunbeams to the leaf, values near 1 indicate leaves perpendicular to the sun. Analogous movement patterns have been published for other Malvaceae species, such as *M. multiflora* (synonym *L. cretica*) [28] and *E. rotundifolia* (synonym *M. rotundifolium*) [29], as well as cotton [39]. *Capparis spinosa* presents a similar leaf movement for some hours during the day according to the stem azimuth and the side of the stem where leaves are situated, yet the overall movement phenomenon is far more complex [9]. At the opposite end of the fully diaheliotropic movement, the mallow of the present study reset leaf solar tracking when influenced by near-zero temperatures. The December measurement was performed under 5 °C and, moreover, following a 6-day period of almost zero temperatures at the end of November (Figure 1). At this measurement, steady values of leaf inclination and azimuth were recorded throughout the day, denoting completely horizontal leaves. To the best of our knowledge, this is the first reference for the arrest of leaf movement due to low temperatures; until now only overcast days have been documented to induce a horizontal reorientation of diaheliotropic leaves [28]. An intermediate movement pattern was recorded in the March measurement when the plants had started recovering from the 10-day February freezing event. With temperature rise, the solar tracking was partially restored, with leaf inclination following the sun’s inclination but with a larger deviation of up to 40°, compared to the near-zero deviation during May and June measurements. Because of the close tightening of leaf azimuth with the sun’s, in March measurement the cos(i) was also intermediate and stable around 0.75. Noteworthy here is the fact that the leaves of the March measurement were the new ones developed after the early February freezing episode. Leaf age may play a role in the not-fully diaheliotropic movement pattern recorded in March. This difference in profile from the subsequent measurements may be ascribed to leaf age, recovery from the February freezing event, or both. Albeit the different explanations that may hold for the March movement, the arrest of diaheliotropic leaf movement during December occurs in mature leaves.

The above-mentioned movement pattern was predominantly ascribed to a temperature effect. One may argue that other fluctuating parameters, i.e., plant/leaf age, light intensity, and/or daylight hours may also play a role. Concerning leaf age, we have considered its possible contribution to the March measurement. Nevertheless, we have to mention that the measurements of December and June were performed on leaves of the same age (~4 months old); the leaves that were present on the plant in December had emerged in September, while the leaves of June were the ones that emerged during the February adverse period. Same-aged leaves in these two measurements followed different movement profiles (Figure 2). This fact in our opinion limits the possible interference of leaf age on the movement dynamics. Light intensity shows some fluctuation along seasons; however, we performed our measurements on completely clear days with the maximum possible light intensities. According to Greer et al. [27], the congeneric *M. parviflora* with a very similar to *M. sylvestris* diaheliotropic diurnal profile showed saturation of tracking rate at 1300 μmol m^−2^ s^−1^. Virtually, the tracking rate did not change much above 1000 μmol m^−2^ s^−1^. Since the incident to leaf PPFD in all our measurements was well above 1000 μmol m^−2^ s^−1^ (Figure 5b) we may argue that light intensities were already high enough and similar for all of our measurements diminishing the possibility to play an important role in such different movement profiles encountered. To the best of our knowledge, there are no available data in the literature on the influence of daylight length on movement profile. The probable reason for that may be the lack of work on the seasonal fluctuation of heliotropic movements. Only, in the laboratory experiment of Greer et al. [27] there is a reference to the effect of exposure time to a directional light on *Malva parviflora* leaf angle. Their results demonstrated that this plant performed a complete shift from the horizontal to an almost vertical to the light beam position after only 400 min (6.6 h) at light intensities like ours, and then remained unchanged. The daylight length in mid-December and mid-June at the latitude of our work’s location is much longer than this “saturation” level (9.24 h and 15 h, respectively). Noteworthy here is the fact that one of our incomplete measurements was performed on the 19th of November of a similar day length (9.52 h) to the December 14th of Figure 2, yet a few days before the near-zero period. While the December measurement showed the arrest of leaf movement, the November 19th measurement showed no such arrest. Consequently, these findings point towards the absence of interference of daylight hours with the movement pattern of mallow.

The main advantage of diaheliotropism is the maximization of light interception by the leaf lamina resulting in higher photosynthetic rates, given that other environmental conditions are favorable. If we consider the combined results of movement, PAR, and ETR in the non-stress conditions of autumn and spring, solar tracking increased PAR incident in the leaf and thereby photosynthetic electron transport (Figure 5b,c). When the low temperature started influencing the movement, intercepted PAR decreased, as well as ETR. Pre-dawn leaf temperature fell below zero at the end of November and during December exerting a negative influence on the maximum PSII efficiency, as illustrated in Figure 5d. The predawn decline of Fv/Fm at this period indicates a persistent downregulation of PSII function. According to Verhoeven et al. [38] the sustained photoinhibitory depression of Fv/Fm during the night is associated with the retention of high amounts of zeaxanthin and antheraxanthin, implying a photoprotective strategy that primes the photosynthetic machinery for non-photochemical quenching at dawn. Several works with *M. neglecta* demonstrate that the engagement of the xanthophyll cycle confers an efficient photoprotection, yet the thermal quenching of the excess energy by this mechanism reduces the photochemical activity of PSII [38,40]. After a temperature rise, either experimentally or naturally as the spring progresses, a disengagement of xanthophylls occurs and simultaneously a recovery of PSII function through changes in other aspects of the photosynthetic process [41]. At midday, the mallow leaves of the present study experienced leaf temperatures always above 10 °C, even in days of below zero pre-dawn temperatures, a fact that favored the PSII operating efficiency, φPSII, measured at noon. 

The above-mentioned works with *M. neglecta* behavior under low temperatures do not consider the role of solar tracking in modifying the photosynthetic responses. During clear days with near-zero temperatures as experienced by *M. sylvestris* of the present study, photoinhibitory conditions prevail. Very low temperature reduces the rates of photosynthetic biochemistry, whereby the photochemical quenching of the absorbed energy. Simultaneously, high radiation on leaf lamina results in over-excitation and possibly photo-oxidation of the reaction centers [42,43]. The synergistic effect of these processes is the imbalance between energy supply and energy utilization leading to photoinhibition. Apparently, this process would be exacerbated by diaheliotropism, which would maintain the input of photons into the photosynthetic cells at a constant level [3]. It is conspicuous that if mallow plants during the December low-temperature period were continuing to closely follow their diaheliotropic movement pattern they would further enhance over-excitation, especially in the morning. Indeed, this problem is more intense in the first morning hours after sunrise, when minimum daily temperatures appear, posing a strong imbalance between dark and light reactions of photosynthesis. Evidently, in such periods mallow leaves arrest movement, avoiding the interception of higher light intensities. We hypothesize that this arrest of movement serves as a protection mechanism from photo-damage during high light and low-temperature conditions. Consequently, the differential leaf movement pattern of *M. sylvestris* may be characterized as opportunistic, since it is used under favorable conditions for the maximization of the intercepted energy, but it is canceled under stress conditions to avoid overexcitation of the photosynthetic machinery.

## 5. Conclusions

*Malva sylvestris* is a diaheliotropic species, which closely follows the sun through both leaf inclination and leaf azimuthal changes. This pattern was followed in periods of favorable environmental conditions to maximize the radiation incident on the leaf lamina. Nevertheless, under near-zero temperature the movement was arrested to protect the photosynthetic apparatus and the relevant physiological processes from photoinhibition and photo-damage, resulting in lower PAR interception and ETR. During these adverse periods, below-zero leaf temperature at pre-dawn was associated with a pronounced decline in the maximum PSII photochemical efficiency. Additionally, during the 10-day freezing event, phenological modifications were recorded, notably leaf shedding and a decrease in the mean area of the remaining leaves; only new leaves of comparatively lower chlorophyll content were present on the plants. The rise in temperature in March triggered a transition phase during which the diaheliotropic movement was partially restored; likewise, the physiological function of the photosynthetic apparatus. The daily and seasonal profiles of Ψ were synergistically shaped by climate and leaf diaheliotropism. The findings of the present study clearly demonstrated that leaf movement in common mallow is a dynamic response to the prevailing temperatures and report for the first time that near-zero temperatures arrest solar tracking.

## Data Availability

The data is available from the authors upon request.

## References

[1] Forterre Y. (2013). Slow, Fast and Furious: Understanding the Physics of Plant Movements. J. Exp. Bot..

[2] Darwin C., Darwin F. (2009). The Power of Movement in Plants.

[3] Koller D. (2000). Plants in Search of Sunlight. Advances in Botanical Research.

[4] Kao W.-Y., Tsai T.-T. (1998). Tropic Leaf Movements, Photosynthetic Gas Exchange, Leaf Δ13C and Chlorophyll a Fluorescence of Three Soybean Species in Response to Water Availability. Plant Cell Environ..

[5] Rakocevic M., Müller M., Matsunaga F.T., Neumaier N., Farias J.R.B., Nepomuceno A.L., Fuganti-Pagliarini R. (2018). Daily Heliotropic Movements Assist Gas Exchange and Productive Responses in DREB1A Soybean Plants under Drought Stress in the Greenhouse. Plant J..

[6] Jiang C.-D., Gao H.-Y., Zou Q., Jiang G.-M., Li L.-H. (2006). Leaf Orientation, Photorespiration and Xanthophyll Cycle Protect Young Soybean Leaves against High Irradiance in Field. Environ. Exp. Bot..

[7] Jiang M., Guo K., Wang J., Wu Y., Shen X., Huang L. (2023). Current Status and Prospects of Rice Canopy Temperature Research. Food Energy Secur..

[8] Xu D.-Q., Chen Y., Chen G.-Y. (2015). Light-Harvesting Regulation from Leaf to Molecule with the Emphasis on Rapid Changes in Antenna Size. Photosynth. Res..

[9] Levizou E., Kyparissis A. (2016). A Novel Pattern of Leaf Movement: The Case of *Capparis spinosa* L.. Tree Physiol..

[10] Zhu C.G., Chen Y.N., Li W.H., Chen X.L., He G.Z. (2015). Heliotropic Leaf Movement of *Sophora alopecuroides* L.: An Efficient Strategy to Optimise Photochemical Performance. Photosynthetica.

[11] Habermann G., Machado S.R., Guimarães V.F., Rodrigues J.D. (2008). Leaf Heliotropism in Styrax Camporum Pohl from the Brazilian Cerrado: Distinct Gas Exchange and Leaf Structure, but Similar Leaf Temperature and Water Relations. Braz. J. Plant Physiol..

[12] Pastenes C. (2004). Leaf Movements and Photoinhibition in Relation to Water Stress in Field-Grown Beans. J. Exp. Bot..

[13] Bielenberg D.G., Miller J.D., Berg V.S. (2003). Paraheliotropism in Two Phaseolus Species: Combined Effects of Photon Flux Density and Pulvinus Temperature, and Consequences for Leaf Gas Exchange. Environ. Exp. Bot..

[14] Fu Q.A., Ehleringer J.R. (1989). Heliotropic Leaf Movements in Common Beans Controlled by Air Temperature 1. Plant Physiol..

[15] Kao W.-Y., Forseth I.N. (1991). The Effects of Nitrogen, Light and Water Availability on Tropic Leaf Movements in Soybean (*Glycine max*). Plant Cell Environ..

[16] Arena C., Vitale L., De Santo A.V. (2008). Paraheliotropism in *Robinia pseudoacacia* L.: An Efficient Strategy to Optimise Photosynthetic Performance under Natural Environmental Conditions. Plant Biol..

[17] Inamullah, Isoda A. (2005). Adaptive Responses of Soybean and Cotton to Water Stress II. Changes in CO _2_ Assimilation Rate, Chlorophyll Fluorescence and Photochemical Reflectance Index in Relation to Leaf Temperature. Plant Prod. Sci..

[18] Huang W., Zhang J.-L., Zhang S.-B., Hu H. (2014). Evidence for the Regulation of Leaf Movement by Photosystem II Activity. Environ. Exp. Bot..

[19] Zhang Y.L., Zhang H.Z., Feng G.Y., Tian J.S., Zhang W.F. (2009). Leaf Diaheliotropic Movement Can Improve Carbon Gain and Water Use Efficiency and Not Intensify Photoinhibition in Upland Cotton (*Gossypium hirsutum* L.). Photosynthetica.

[20] Thanisawanyangkura S., Sinoquet H., Rivet P., Cretenet M., Jallas E. (1997). Leaf Orientation and Sunlit Leaf Area Distribution in Cotton. Agric. For. Meteorol..

[21] Sailaja M.V., Rama Das V.S. (1996). Leaf Solar Tracking Response Exhibits Diurnal Constancy in Photosystem II Efficiency. Environ. Exp. Bot..

[22] Ehleringer J.R., Forseth I. (1980). Solar Tracking by Plants. Science.

[23] Kornyeyev D., Logan B.A., Allen R.D., Holaday A.S. (2005). Field-Grown Cotton Plants with Elevated Activity of Chloroplastic Glutathione Reductase Exhibit No Significant Alteration of Diurnal or Seasonal Patterns of Excitation Energy Partitioning and CO_2_ Fixation. Field Crops Res..

[24] Xu F., Guo W., Wang R., Xu W., Du N., Wang Y. (2009). Leaf Movement and Photosynthetic Plasticity of Black Locust (*Robinia Pseudoacacia*) Alleviate Stress under Different Light and Water Conditions. Acta Physiol. Plant..

[25] Ceccanti C., Landi M., Guidi L., Pardossi A., Incrocci L. (2022). Seasonal Fluctuations of Crop Yield, Total Phenolic Content and Antioxidant Activity in Fresh or Cooked Borage (*Borago officinalis* L.), Mallow (*malva sylvestris* L.) and Buck’s-Horn Plantain (*Plantago coronopus* L.) Leaves. Horticulturae.

[26] Fisher F.J.F., Ehret D.L., Lister G.R., Hollingdale J. (1989). Light Quality and Sun Tracking in Malva Neglecta. Can. J. Bot..

[27] Greer D.H., Thorpe M.R. (2009). Leaf Photosynthetic and Solar-Tracking Responses of Mallow, Malva Parviflora, to Photon Flux Density. Plant Physiol. Biochem..

[28] Schwartz A., Koller D. (1986). Diurnal Phototropism in Solar Tracking Leaves of *Lavatera cretica*. Plant Physiol..

[29] Forseth I.N., Ehleringer J.R. (1982). Ecophysiology of Two Solar Tracking Desert Winter Annuals. Oecologia.

[30] Prichard J.M., Forseth I.N. (1988). Rapid Leaf Movement, Microclimate, and Water Relations of Two Temperate Legumes in Three Contrasting Habitats. Am. J. Bot..

[31] Forseth I., Ehleringer J.R. (1980). Solar Tracking Response to Drought in a Desert Annual. Oecologia.

[32] Lang A.R.G. (1973). Leaf Orientation of a Cotton Plant. Agric. Meteorol..

[33] Lichtenthaler H.K., Wellburn A.R. (1983). Determinations of Total Carotenoids and Chlorophylls a and b of Leaf Extracts in Different Solvents. Biochem. Soc. Trans..

[34] Murchie E.H., Lawson T. (2013). Chlorophyll Fluorescence Analysis: A Guide to Good Practice and Understanding Some New Applications. J. Exp. Bot..

[35] Goswami A.K., Maurya N.K., Goswami S., Bardhan K., Singh S.K., Prakash J., Pradhan S., Kumar A., Chinnusamy V., Kumar P. (2022). Physio-Biochemical and Molecular Stress Regulators and Their Crosstalk for Low-Temperature Stress Responses in Fruit Crops: A Review. Front. Plant Sci..

[36] Zhao Y., Han Q., Ding C., Huang Y., Liao J., Chen T., Feng S., Zhou L., Zhang Z., Chen Y. (2020). Effect of Low Temperature on Chlorophyll Biosynthesis and Chloroplast Biogenesis of Rice Seedlings during Greening. Int. J. Mol. Sci..

[37] Muller O., Stewart J.J., Cohu C.M., Polutchko S.K., Demmig-Adams B., Adams W.W. (2014). Leaf Architectural, Vascular and Photosynthetic Acclimation to Temperature in Two Biennials. Physiol. Plant..

[38] Verhoeven A.S., Adams W.W., Demmig-Adams B. (1999). The Xanthophyll Cycle and Acclimation of Pinus Ponderosa and Malva Neglecta to Winter Stress. Oecologia.

[39] Ehleringer J.R., Hammond S.D. (1987). Solar Tracking and Photosynthesis in Cotton Leaves. Agric. For. Meteorol..

[40] Verhoeven A.S., Adams W.W., Demmig-Adams B. (1996). Close Relationship between the State of the Xanthophyll Cycle Pigments and Photosystem II Efficiency during Recovery from Winter Stress. Physiol. Plant..

[41] Adams W.W., Demmig-Adams B., Rosenstiel T.N., Ebbert V. (2001). Dependence of Photosynthesis and Energy Dissipation Activity upon Growth Form and Light Environment during the Winter. Photosynth. Res..

[42] Ivanov A.G., Sane P., Hurry V., Król M., Sveshnikov D., Huner N.P.A., Öquist G. (2003). Low-Temperature Modulation of the Redox Properties of the Acceptor Side of Photosystem II: Photoprotection through Reaction Centre Quenching of Excess Energy. Physiol. Plant..

[43] Nilsen E.T., Arora R., Upmanyu M. (2014). Thermonastic Leaf Movements in Rhododendron during Freeze–Thaw Events: Patterns, Functional Significances, and Causes. Environ. Exp. Bot..

